# The Rh blood group system and its role in alloimmunization rate among sickle cell disease and sickle thalassemia patients in Iran

**DOI:** 10.1002/mgg3.1614

**Published:** 2021-02-06

**Authors:** Mohammad Ali Jalali Far, Arezoo Oodi, Naser Amirizadeh, Mahshid Mohammadipour, Bijan Keikhaei Dehdezi

**Affiliations:** ^1^ Blood Transfusion Research Center High Institute for Research and Education in Transfusion Medicine Tehran Iran; ^2^ Thalassemia & Hemoglobinopathy Research Center, Research Institute of Health Ahvaz Jundishapur University of Medical Sciences Ahvaz Iran

**Keywords:** alloimmunization, RH genotype, Rh phenotype, sickle cell disease, sickle thalassemia

## Abstract

**Introduction:**

The alloimmunization following blood transfusion can be life‐threatening. The Rh alloantibodies are one of the most common causes contributing to alloimmunization. This study aimed to evaluate the rate and causes of alloimmunization and to determine the Rh phenotypes and genotypes among sickle cell disease (SCD) and sickle thalassemia (Sβ).

**Materials and Methods:**

Our study included 104 SCD and Sβ patients referring to Baghaei 2 Hospital of Ahvaz in 2019 using a non‐random simple sampling method. The blood samples were collected for Rh phenotypes, alloantibody screening and identification, and molecular tests. The SSP‐PCR and RFLP methods with the Pst 1 enzyme were used.

**Results:**

The alloimmunization rate was 9.6% and 13.2% based on immunohematological tests and medical records, respectively. The main alloantibodies (90%) were anti‐Rh, and 40% of the patients had multiple alloantibodies. A significant correlation was found between gender and alloimmunization. The phenotypes of DCce (37.5%), DCcEe (24%), Dce (20.2%), and dce (5.8%) and genotypes of R1r (25%), R1R2 (20.2%), R1R1 (18.3%), and R1R0 (10.6%) were the most prevalent. The R1R2 was a frequent genotype in Sβ.

**Conclusion:**

R0r′ and R1R0 genotypes were limited to our population in Iran. Due to the differences in RH genotypes between our population and others, the blood transfusion from other ethnicities increased our total alloimmunization rate.

## Graphical abstract

- The alloimmunization is the problem in 9.6% of sickle cell disease (SCD) and sickle thalassemia patients.
- The increase in the rate of alloimmunization was due to Rh alloantibodies.
- The Rh genotypes were different among sickle cell disease and sickle thalassemia patients than other populations and R1r′ and R0R1 genotypes were limited to our population.

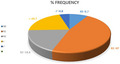

## 1 INTRODUCTION

The sickle cell disease (SCD) and other hemoglobinopathies are one of the main health problem in Iran. The native and main region of SCD is southwest of Iran. Advances in medical sciences have not yet been able to replace the unique role of blood transfusion in managing complications and saving the lives of SCD patients and other transfusion‐dependent hemoglobinopathies. As a supportive treatment strategy, blood transfusion is still saving the lives of many patients (Chou, 2013; Davies & Roberts‐Harewood, 1997; Hood et al., 2019; Wahl & Quirolo, 2009). However, the occurrence of alloimmunization following repeated blood transfusions is one of the most severe complications in these patients. In addition to complicating the clinical condition of these patients and imposing huge costs on them for their medical management, there are concerns for these patients, their physicians, and blood transfusion services in terms of finding compatible blood in subsequent blood transfusions, more alloimmunizations, and production of multiple alloantibodies. It has been shown that after the first alloimmunization, patients will be prone to develop multiple alloantibodies (Gehrie et al., 2017; Yazdanbakhsh et al., 2012). Depending on the population, different ethnicities, and the study region, the rate of alloimmunization in different studies is very different and varies from 4% to 42% (Meda et al., 2014; Nickel et al., 2015). On the other hand, alloimmunization in sickle cell patients is higher than that in thalassemia major patients in terms of both prevalence rate and multiple alloantibodies (Chou et al., 2012, 2013), calling for more attention to study the identification of the alloimmunization rate and its reason among the SCD. The most common alloantibodies reported in SCD and thalassemia were anti‐ Rh antigens and anti‐Kell blood group system (Al‐Mousawi et al., 2015; Alkindi et al., 2017; Ameen et al., 2009; Bashawri, 2007; Vafaei & Keikhaei Dehdazi, 2017).

The Rh blood group system is the most polymorphic blood group antigen, and the most immunogenic after the ABO blood group, and it is the most clinically significant blood group in blood transfusion (Avent & Reid, 2000; Westhoff, 2004). Depending on the study region, population, and even ethnicity, the Rh antigens’ distribution has been different in different studies. The differences between blood donors and recipients other than ABO blood grouping are among the leading causes of alloimmunization in blood transfusion‐dependent patients (Chou, 2013). A study on the rate and causes of alloimmunization as well as determination of prominent phenotype and genotypes of the Rh blood group system in SCD and sickle thalassemia (Sβ) patients in Ahvaz as one of the main areas of SCD and Sβ outbreak can be helpful in appropriate management of patients and prevention of their alloimmunization.

## 2 MATERIALS AND METHODS

### 2.1 Ethic statement

The study protocol was approved by the local medical ethics committee of the Higher Educational Institute of Blood Transfusion, Tehran, Iran. All participants or their parents read and signed written informed consent before enrollment. They were also informed that all clinical data would be used only for scientific and not for commercial purposes. All clinical information and medical histories were collected at the Thalassemia & Hemoglobinopathy Research Center, Research Institute of Health, Ahvaz Jundishapur University of Medical Sciences.

### 2.2 Study design

This was a descriptive cross‐sectional study in which all samples were collected using non‐random simple sampling method. Our study population included all patients with SCD and Sβ referring to Baqaei 2 Hospital in Ahvaz in 2019. The blood samples were collected in two ethylenediaminetetraacetic acid (EDTA)‐containing tubes as anticoagulant from each patient for serological and molecular tests. The demographic details and information about the blood transfusion, pregnancy, and previous alloimmunization history were collected from their medical records of patients. All immunohematological tests including the Rh main antigens typing, antibody screening, and antibody identification were performed on fresh blood samples based on standard tube methods. For molecular experiments and DNA extraction, the buffy coat was separated on the same day of blood collection, and in case DNA extraction was not possible, the samples were kept at refrigerator temperature up to 48 hours. After DNA extraction, the samples were stored at −25°C until the experiments started.

### 2.3 Inclusion and exclusion criteria

All patients with SCD or Sβ signed the informed consent for participation in the study and did not have a history of blood transfusion during sample collection. If the patients had a disease other than SCD or Sβ or had a history of blood transfusions at the time of sample collection, they would be excluded from the study.

### 2.4 Serological methods for determining the blood group based on the Rh system

The commercial antisera against D, C, c, and E antigens (IMMUNDIAGNOSTICA GmbH, Germany) were used for the Rh blood group typing. The test was performed using a standard serological tube method based on hemagglutination following the manufacturer's instruction.

#### 2.4.1 Antibody screening and identification

For antibody screening, the patients’ plasma was incubated with a commercial triple panel cell (Panocell, USA). The test was done according to standard serological methods (Immediate spin, Room Temperature, and Anti‐Human Globin phases). If the antibody screening test was positive, the antibody identification was performed using 11 panels (Panocell, USA). In case there were multiple antibodies, a selective panel with 20 cells (Panocell, USA) and specific antisera was used for ruling out or confirming the presence of specific alloantibodies.

### 2.5 Molecular tests

#### 2.5.1 DNA extraction

We used a commercial kit for extraction of genomic DNA from the patients’ buffy coat (Yekta Tajhiz Azma, Iran). All steps were performed following the manufacturer's instructions. The purity and concentration of the obtained DNA were determined using Nanodrop (Thermo Fisher Scientific, USA). The DNA was then stored at −25°C until molecular tests were performed.

#### 2.5.2 Single specific primer‐polymerase chain reaction (SSP‐PCR) for evaluation of the Rhesus box

The microtube's content was as follows: 12.5 μl of Commercial 2X Master Mix (Yekta Tajhiz Azma, Iran), at final concentration of 500 nanomolar for each of the Forward primer (5′‐TGAGCCTATAAAATCCAAAGCAAGTTAG‐3′) and Reverse primer (5′‐CCTTTTTTTGTTTGTTTTTGGCGGTGC‐3′), the concentration of DNA added was 40 ng/μl, and the final volume of the sample was increased to 25 μl by the addition of sterile distilled water. The thermocycling profile is given in Table 1. In the presence of the Rhesus box, the 2278 bp bands can be seen on electrophoresis on 1% agarose gel (Sigma, USA) with 1 Kb Ladder (Yekta Tajhiz Azma, Iran).

**TABLE 1:** The thermocycling profile of the SSP‐ PCR of *Rhesus* box (Primer concentration: 500 nano mol, DNA concentration: 40 ng)

Step	Temperature/°C	Action	Time/s	Cycle. no
1	95	Denaturation	120	1
2	95	Denaturation	30	35
	64	Annealing	30	
	68	Extension	180	
3	72	Extension	300	1

#### 2.5.3 Rhesus PCR‐RFLP (Restriction Fragment Length Polymorphism) for determination of *RHD* gene zygosity using Pst 1 enzymatic digestion

**FIGURE 1:**
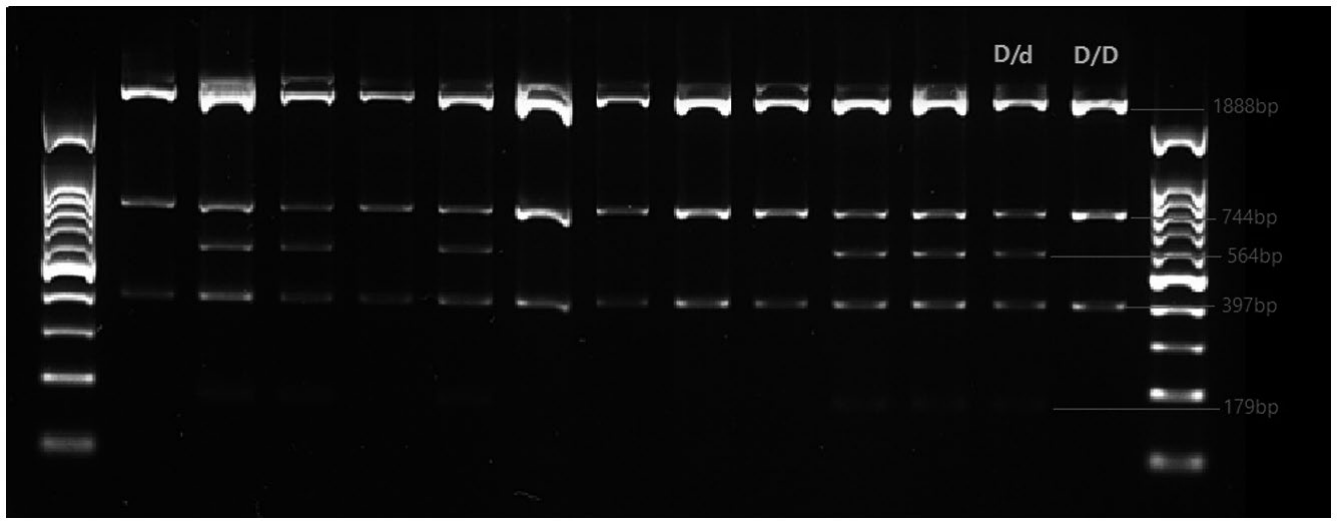
RFLP using Pst I enzyme/heterozygous and homozygous form of* RHD* gene using enzymatic cleavage pattern. There are 3 Pst I sites in the amplicon in D‐negative haplotypes resulting in fragments of 1888, 564, 397, and 179 bp. The downstream Rhesus box of D‐positive haplotypes lacks 1 Pst I site, resulting in fragments of 1888, 744, and 397 bp. *RHD 1*/*RHD 2* heterozygotes show both fragments of 744 and 564 bp. The 564‐bp fragment appears weaker because Pst I does not cut heterodimers and amplifies the upstream Rhesus box of D‐positive haplotypes

To enhance the precision of our study and to ensure about the SSP‐PCR for Rhesus box analysis, we performed the RFLP for *RHD* gene zygosity. The amplified PCR product was obtained using oligonucleotide primers (Rez 7 and Rnb3) based on the study of Khosroshahi et al. (2019). The amplicons obtained from the PCR step were enzymatically digested using Pst I enzyme (Thermo Fisher Scientific, USA) following the manufacturer's instructions. The product obtained by enzymatic digestion was electrophoresed on 2% agarose gel. In D‐negative haplotypes, there are three Pst I sites in the amplicon resulting in fragments of 1888, 564, 397, and 179 bp. In D‐positive haplotypes, fragments of 1888, 744, and 397 bp are seen. *RHD 1*/*RHD 2* heterozygotes show both fragments of 744 and 564 bp (Figure 1).

### 2.6 Statistical analysis

Statistical analysis was performed using SPSS (Statistical Package for the Social Sciences) software version 21. The data were presented using descriptive statistics including mean ± *SD*, frequencies, and percentages. The Chi‐square analysis was employed to compare the alloimmunization rate between our study and other studies and haplotype/genotype frequencies and percentages between groups too.

## 3 FINDINGS

The samples were obtained from 104 patients in the age range from 2 to 67 years. Of all patients, 93% were of Arab ethnicity, and more than half lived in Ahvaz. As far as the disease was concerned, 86% of the patients were sickle cell patients, and 14% were Sβ patients. Sixty percent of our population were hospitalized, and the rest were outpatients. In terms of gender distribution, 59% of the study population were male, and 41% were female (Table 2).

**TABLE 2:** The frequency of different variables among our population

Variable	Subdivide	No.	Percent %
Age group	<18	37	35.9
	18–34	52	50.5
	>35	14	13.6
Sex	Male	42	40.8
	Female	61	59.2
Disease	Sickle cell disease	87	86.1
	Sickle thalassemia	14	13.9
Admission type	Outpatients	62	60.2
	Hospitalized	41	39.8
ABO blood group	O	31	34.1
	A	27	29.7
	B	28	30.8
	AB	5	5.5
Alloimmunization	Yes	12	13.2
History	No	79	86.8
Rh blood group (serologic)	D	97	93.3
	C	85	81.7
	c	82	78.8
	E	33	31.7
	e	100	96.2

In the antibody screening test, 10 patients (9.6%) had alloantibodies. No autoantibodies were observed in any of the studied patients. Antibody identification in our study population showed that 90% of alloantibodies were anti‐Rh, and 40% of patients had developed more than one alloantibody. The all alloimmunized patients were SCD. There was a significant correlation of alloimmunization with gender and history of blood transfusion reactions, while no such correlation was found with age, type of admission, pregnancy history, and ABO blood group.

In our study population, molecular analysis of Rhesus box showed that 57 patients (54.8%) had no Rhesus box, and 47 patients (45.2%) were positive in this regard. In the RFLP study and after Pst I enzymatic digestion, it was found that seven (6.7%) patients were d/d, 40 patients (38.5%) were D/d, and 54.8% were D/D patients. Figure 1 shows the results of the RFLP test.

**FIGURE 2:**
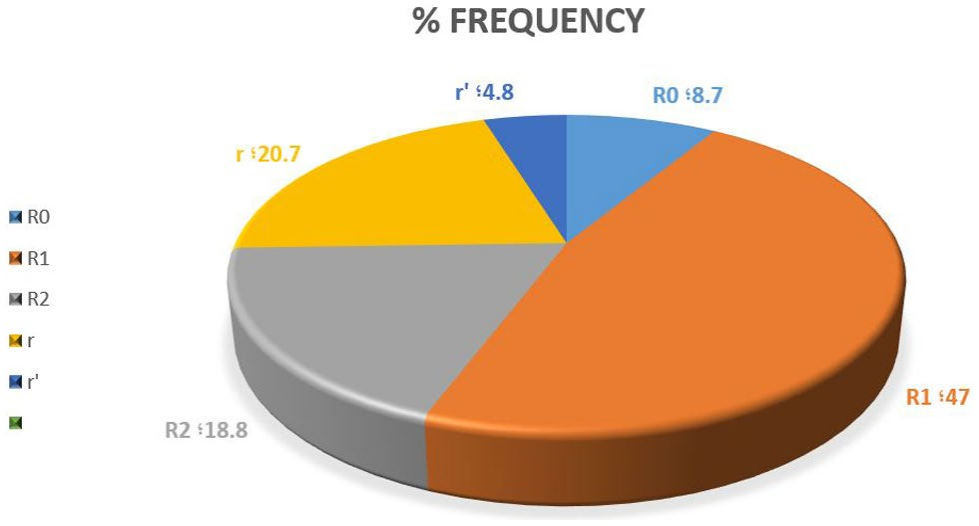
The frequency of different RH haplotypes among our population based on the immunohematological method

The prevalence of Rh haplotype is shown in Figure 2 using serological and molecular studies. Genotype R1 (DCe) with 47% had the most common haplotype, and r′ with 4.8% had the lowest frequency in our study population (Figure 2).

The most common genotypes in the Rh blood group system in our patients were R1r, R1R2, and R1R1, with 25%, 20.2%, and 18.3%, respectively (Table 3).

**TABLE 3:** The RH blood group genotype frequency among our population

*RH* genotype	Population no. (%)		Total no. (%)
	Sickle cell disease	Sickle thalassemia	104 (100)
*R1r*	23 (22.1)	3 (2.9)	26 (25)
*R2r′*	5 (4.8)	2 (1.9)	7 (6.7)
*R1R2*	16 (15.4)	5 (4.8)	21 (20.2)
*R0R1*	11 (10.6)	0 (0)	11 (10.6)
*R0r*	1 (1)	0 (0)	1 (1)
*R2R2*	3 (2.9)	1 (1)	4 (3.8)
*R1R1*	18 (17.3)	1 (1)	19 (18.3)
*rr*	5(4.8)	1 (1)	6 (5.8)
*rr′*	0 (0)	1 (1)	1 (1)
*R0R0*	3 (2.9)	0 (0)	3 (2.9)
*R1r′*	2 (1.9)	0 (0)	2 (1.9)
*R2r*	3 (2.9)	0 (0)	3 (2.9)

R1R1 genotype was common among SCD. In comparison, R1R2 showed a higher prevalence of sickled thalassemia patients. Due to the smaller number of Sβ patients, it was impossible to examine the statistical correlation between genotypes.

## 4 DISCUSSION

Blood transfusions in patients suffering from diseases such as sickle cell anemia, thalassemia, and sickle cell thalassemia, as a therapeutic and supportive approach can be beneficial and life‐saving, but the occurrence of alloimmunization in these patients can cause serious problems for them, their physicians, and blood transfusion services. Our study showed an alloimmunization rate of 9.6% based on the serological tests, while the rate of alloimmunization reached 13.8% in their medical records. That difference was due to alloantibodies that were undetectable at the time of our study. According to our results, 90% of alloantibodies were anti‐Rh blood group system, and 40% of alloimmunized patients had more than one alloantibody. All of our alloimmunized patients were those suffering from SCD, and it was due to small sample size of Sickle thalassemia and different RH genotype between SCD and Sβ. There have been many alloimmunization studies focusing on transfusion‐dependent patients. Because the SCD is more limited to specific geographical areas and ethnicities than thalassemia disease, most of these studies have been conducted on thalassemia patients. In our study, 92% of our patients were of Arab ethnicity, which confirms the prevalence of certain diseases among certain ethnicities. In the study of Mohammadi Maram et al., 0.4% of patients referring to Imam Hospital in Tehran had alloantibodies, and 61.5% of these antibodies were anti‐Rh blood group system (Mohamadimaram et al., 2019). The reason for the low prevalence was that their population did not include transfusion‐dependent patients. In other studies in Iran on transfusion‐dependent thalassemia patients, the rates of alloimmunization were 10.9%, 4%, 63.4%, 31.57%, and 28.7%. The prevalence of anti‐Rh blood group alloantibodies was 58.3%, 25%, 49.9%, and 55%, respectively, and in the Kangiwa et.al study, the prevalence of alloimmunization was not mentioned (Chou et al., 2013; Eghbali et al., 2019; Hiradfar et al., 2010; Kangiwa et al., 2015; Natukunda et al., 2010; Rees et al., 2010; Ware et al., 2017). In a study by Vafaei et al. on sickle cell and thalassemia patients in Ahvaz, 7.1% of the patients had alloantibodies, 40% of alloimmunized patients had anti‐Rh, and 50% had anti‐Kell alloantibodies (Vafaei & Keikhaei Dehdazi, 2017). This study was significantly different from our study in terms of both alloimmunization prevalence and type of alloantibody, indicating an increase in alloimmunization and the development of alloantibodies against the Rh system. The alloimmunization and the type of alloantibodies among different part of Iran are summarized in Table 4. The reason for the decrease in the prevalence of anti‐Kell alloantibodies in our study compared with that in Vafaei et al. is the start of screening all RBC units of blood donors for the Kell blood group and transfusion of the Kell‐matched packed RBC to transfusion‐dependent patients. The increase in the rate of alloimmunization and anti‐ Rh alloantibodies can be attributed to ignoring the Rh blood group system and blood donors’ different ethnicities. Although most reports have confirmed and recommended partially or totally matched blood transfusion for SCD and other blood‐dependent patients, given the financial resource limitations and the large number of blood‐dependent patients in our region, this protocol is not fully applicable there. Previous studies have emphasized the importance of this issue in the development of alloimmunization (Chou, 2013; Eghbali et al., 2019; Rees et al., 2010). Due to the great diversity in the Rh blood group system, even in cases of blood transfusion compatible with the main Rh antigens, the rate of alloimmunization due to the presence of Rh variants is still high (Ware et al., 2017). The prevalence of alloimmunization in neighboring countries and other populations is highly variable. In Iraqi Kurdistan, for example, Al‐Mousawi et al. reported an alloimmunization rate of 5.2% in beta‐thalassemia patients and 2.2% in SCD, 80% of whom had anti‐Rh system alloantibodies (Al‐Mousawi et al., 2015). That report is the only study in which the prevalence of alloimmunization was lower than that in our study. The reason for this low prevalence could be the use of donated blood within the same ethnicities and genetic background, but as in our study, Rh alloantibodies were still the most important cause of alloimmunization. In other studies, the prevalence of alloimmunization was higher. In Alkindi et al.’s study in Oman, for example, the alloimmunization prevalence in SCD and thalassemia patients was 31.6% and 20%, respectively, and 85% of alloantibodies were against the Rh and Kell antigens (Alkindi et al., 2017). Bashawri et al. in Saudi Arabia reported an alloimmunization prevalence of 13.7%, and 56.3% of alloimmunization was anti‐Rh system alloantibodies (Bashawri, 2007). Ameen et al. in Kuwait reported a 63.6% alloimmunization rate in SCD patients who received standard ABO‐ and D‐matched non‐leukoreduced blood. In the second group of their study, which was transfused with leukoreduced RBCs matched for ABO, Rh, and K1, the rate was 23.6%, and anti‐Rh alloantibodies were significantely prevalent in both groups (67% in the first group and 86% in the second group) (Ameen et al., 2009). Studies in other countries also reported a variable prevalence between 4% and 42.1% (Chou et al., 2013; Kangiwa et al., 2015; Karafin et al., 2018; Meda et al., 2014; Moreira Jr et al., 1996; Natukunda et al., 2010; Nickel et al., 2015). Consistent with other studies, we found that the prevalence of alloimmunization had a significant correlation with gender, maybe due to more exposure to immunizing events through pregnancy and/or transfusions in females with SCD (Abbas et al., 2013; Ameen et al., 2009; Moreira Jr et al., 1996; Verduin et al., 2012, 2015), which underscores the need for more attention to the occurrence of alloimmunization and taking preventive actions. There are several reasons for these discrepancies in the results regarding the rate of alloimmunization in SCD patients, some of which may be related to the nature of the antigens and their immunogenicity potency, and some to the patient's factors, the discordance of blood group antigen expression on donor, and patient RBCs due to differences between the ethnicities of the patient, and the blood donor is likely the most important contributing factor (Elenga & Niel, 2015; Gehrie et al., 2017; Moreira Jr et al., 1996; Verduin et al., 2012). According to the above findings, the Rh blood group system is greatly important due to its immunogenicity and antigen diversity.

**TABLE 4:** The alloimmunization rate and the type of alloantibodies among different studies in Iran

Author(s)	Population	Region	Alloimmunization rate %	Type and prevalence rate of alloantibodies
Current study	SCD and Sickle thalassemia	Ahvaz	9.6%	Anti‐c: 60%; anti‐ E: 40%; Fy^a^: 30%; Fy^b^:10%; Anti‐Kell: 20%; Anti‐S: 20%
Davoudi‐Kiakalayeh et al. (2017)	190 multi transfused β‐thalassemia major	North of Iran	24.7	Anti –Rh:31 case
				Anti‐Kell: 21, Anti‐Fy^a^: 1
				Anti‐S: 1, anti‐Kp^a^: 3
Moradinasab et al. (2019)	6029 hospitalized patients in of Imam Khomeini general hospital	Tehran	0.5%	Anti‐D (30%), Anti‐E (24%), anti‐K (12%), Anti‐C (10%), Anti‐c (8%)
				Anti‐e (8%), Anti‐Jk^b^ (4%)
				Anti‐s (4%)
Vafaei and Keikhaei Dehdazi (2017)	68 SCD and 72 S/B thalassemia.	Ahvaz	7.1	50% anti‐Kell, 30% anti‐E and 10% anti‐D and 10% unknown
Sarihi et al. (2020)	480 beta thalassemia major patients	Tehran (multicenter)	13.5%	Central region: Anti‐K (25.1%), anti‐E (15.4%), anti‐D (10.8%) and anti‐C (7.7%)
				Northern region: anti‐K (28.4%), anti‐E (22.2%), anti‐D (10.5%) and anti‐C (6.5%)
				Southwest region: anti‐K (25.8%), anti‐E (20.2%), anti‐D (11.2%) and anti‐Kp^a^ (5.6%)
				Southern region: Anti‐K (24.2%), anti‐E (21.2%), anti‐D (9.1%) and anti‐C (9.1%)
				Western area. Anti‐K (37.7%), anti‐D (15.1%), anti‐Kp^a^ (11.3%), anti‐E (9.4%)
Mirzaeian et al. (2013)	385 beta thalassemia major patients	Zahedan	17.9 (allo‐Ab) 5.5 (auto‐Ab)	Undetermined (3.1) Anti‐E (2.6) Anti‐C (1.6) Anti‐Lua (1.6) Anti‐c (1.3) Anti‐Cw (1.3) Anti‐K (1)
Davari and Soltanpour (2016)	49 β‐thalassemia major patients	Zanjan	16.32%	Anti‐K (60) Anti‐E (10) Anti‐c (10) Anti‐Leb (10)

The Rh blood group system is the most immunogenic blood group after ABO and the most polymorphic blood group system (Avent & Reid, 2000; Westhoff, 2004). In our study, 90% of alloantibodies were anti‐Rh blood group system. Although the prevalence of C and c antigens was higher than that in other studies, there was no significant difference. The prevalence of other antigens in this system was similar to that obtained in other studies conducted in Iran. Compared to Vafaei et al., in our study, the prevalence of E antigen was similar to that of Arab donors but lower than that of their SCD population (Ahmadi et al., 2019; Dastjerdi et al., 2018; Tangvarasittichai, 2017; Vafaei & Keikhaei Dehdazi, 2017). Based on our findings, R1r, R1R2, R1R1, and R0R1 were the most common genotypes in the study population. According to data summarized in Table 5, our finding was different from other studies in terms of both the prevalence of RH haplotypes and the order of prevalence (Abdi & Kiani, 2009; Bogui et al., 2014; Keramati et al., 2011; Reid et al., 2012; Shahverdi et al., 2016; Tangvarasittichai, 2017). In addition to the genetic differences of our study population with those of other studies, our different results could be attributed to the high accuracy of our study in determining the zygosity of *the RHD* gene by molecular methods, which increases the accuracy of genotype determination. Given the increasing rate of alloimmunization in the population of Khuzestan province where this study was conducted and the predominance of Rh alloantibodies therein, national and local blood transfusion policies should be adopted to prevent further costs and complexity of these patients’ clinical management.

**TABLE 5:** The RH blood group genotypes among different studies and population

	Genotype (%)																	
Population (Authors)	R1r	R1R2	R1R1	R1r′	R0R1	R2r′	rr	R2R2	R0R0	R2r	R0r	r'r	R1RZ	R2Rz	RzRz	r'r′	r″r′	r″r
Current study	25	20.2	18.3	10.9	10.6	6.7	5.8	3.8	2.9	2.9	1	1	–	–	–	–	–	–
General population of Qazvin, Iran (Ahmadi et al., 2019)	29	15.6	25.8	–	–	–	–	3.4	–	8.8	4.1	1.4	1.2	0.3	–	<0.01	0.6	0.4
Isfahan Repeated blood donors (Dastjerdi et al., 2018)	27.42	16.45	22.26	–	–	–	10	3.55	–	9.68	5.16	1.29	1.61	0.32	–	0.97	0.65	0.32
Blood donors in Khorramabad, Iran (Abdi & Kiani, 2009)	28.2	16.3	26.9	–	–	10.6	5.7	4.3	–	–	3.7	<0.001	0.8	<0.001	–	<0.001	<0.001	0.4
Blood donors in Mashhad, northeast of Iran (Keramati et al., 2011)	31.8	16.5	25	–	–	–	8.3	1.7	–	9.6	4.2	1.3	1	0.4	–	–	0	0.2
Iranian population (Shahverdi et al., 2016)	27.7	14.59	22.38	–	–	–	9.59	2.30	–	10.35	1.78	1.95	0.08	0.01	1.95	0.04	0.05	0.45
Blood donors in Cote d’Ivoire, West Africa (Bogui et al., 2014)	20	7	–	–	–	–	‐	–	–	12.73	65.12	–	–	–	0.17	–	–	–
Caucasian	34.9	13.3	18.5	–	–	–	15.1	2.3	–	11.8	2.1	0.8	0.2	0.1	0.01	Rare	0.05	0.9
Asian	21	4	2	–	–	–	6.8	0.2	–	18.6	45.8	Rare	Rare	Rare	Rare	Rare	Rare	Rare
Black (Reid et al., 2012)	8.5	30	51.8	–	–	–	0.1	4.4	–	2.5	0.3	0.1	1.4	0.4	Rare	0.1	Rare	Rare

The rate of alloimmunization in our study population was lower than that in other studies, which could be due to good management and the use of the Kell‐matched blood for transfusion. Compared to a previous study, the increase in alloimmunization may be due to the anti‐Rh blood group alloantibodies of blood from other provinces. Variation and difference in RH genotype in our study population compared to other populations confirm our hypothesis and highlight the importance of adopting national and local policies to prevent alloimmunization. Because blood transfusion can interfere with immunohematological tests, molecular tests to determine clinically important groups are recommended.

## CONFLICT OF INTEREST

The authors declare no conflict of interests in relation to this study.

## AUTHOR CONTRIBUTIONS

MAJF carried out the serological and molecular analysis and drafting of the manuscript. NA was responsible for project administration, conceptualization, study design and drafted the manuscript. AO conducted the data analysis and molecular methods optimization. MM and BKD were involved in the study design and methodology. All authors read and approved the final version of the manuscript and agree with the order of presentation of the authors.

## Data Availability

The datasets used and/or analyzed during the current study are available from the corresponding author on reasonable request.
